# CMR Native T1 Mapping Allows Differentiation of Reversible Versus Irreversible Myocardial Damage in ST-segment–Elevation Myocardial Infarction: An OxAMI Study (Oxford Acute Myocardial Infarction)

**DOI:** 10.1161/CIRCIMAGING.116.005986

**Published:** 2017-08-10

**Authors:** Dan Liu, Alessandra Borlotti, Dafne Viliani, Michael Jerosch-Herold, Mohammad Alkhalil, Giovanni Luigi De Maria, Gregor Fahrni, Sam Dawkins, Rohan Wijesurendra, Jane Francis, Vanessa Ferreira, Stefan Piechnik, Matthew D. Robson, Adrian Banning, Robin Choudhury, Stefan Neubauer, Keith Channon, Rajesh Kharbanda, Erica Dall’Armellina

**Affiliations:** From the Division of Cardiovascular Medicine, Radcliffe Department of Medicine, John Radcliffe Hospital, University of Oxford, Headley Way, United Kingdom (D.L., A.B., D.V., M.A., G.L.D.M., G.F., S.D., R.W., J.F., V.F., S.P., M.D.R., R.C., S.N., E.D.A.); Department of Cardiovascular Medicine, Oxford Heart Centre, John Radcliffe Hospital, Headley Way, United Kingdom (A.B., K.C., R.K.); and Department of Radiology, Brigham and Women’s Hospital, Boston, MA (M.J.-H.).

**Keywords:** magnetic resonance imaging, microcirculation, myocardial infarction, ventricular remodeling

## Abstract

**Background—:**

CMR T1 mapping is a quantitative imaging technique allowing the assessment of myocardial injury early after ST-segment–elevation myocardial infarction. We sought to investigate the ability of acute native T1 mapping to differentiate reversible and irreversible myocardial injury and its predictive value for left ventricular remodeling.

**Methods and Results—:**

Sixty ST-segment–elevation myocardial infarction patients underwent acute and 6-month 3T CMR, including cine, T2-weighted (T2W) imaging, native shortened modified look-locker inversion recovery T1 mapping, rest first pass perfusion, and late gadolinium enhancement. T1 cutoff values for oedematous versus necrotic myocardium were identified as 1251 ms and 1400 ms, respectively, with prediction accuracy of 96.7% (95% confidence interval, 82.8% to 99.9%). Using the proposed threshold of 1400 ms, the volume of irreversibly damaged tissue was in good agreement with the 6-month late gadolinium enhancement volume (*r*=0.99) and correlated strongly with the log area under the curve troponin (*r*=0.80) and strongly with 6-month ejection fraction (*r*=−0.73). Acute T1 values were a strong predictor of 6-month wall thickening compared with late gadolinium enhancement.

**Conclusions—:**

Acute native shortened modified look-locker inversion recovery T1 mapping differentiates reversible and irreversible myocardial injury, and it is a strong predictor of left ventricular remodeling in ST-segment–elevation myocardial infarction. A single CMR acquisition of native T1 mapping could potentially represent a fast, safe, and accurate method for early stratification of acute patients in need of more aggressive treatment. Further confirmatory studies will be needed.

## Abstract 2

Supplemental Digital Content is available in the text.

Prognosis after acute myocardial infarction (MI) is primarily dictated by the extent of irreversible myocardial injury and by left ventricular (LV) remodeling. Numerous mechanical, macrovascular, microvascular, and biochemical factors are known to contribute to the early myocardial changes after ischemia reperfusion.^1^ Despite the advanced knowledge of the pathophysiology of ischemia reperfusion, the translation of these findings into effective clinical therapies has been limited in the past.^1^ Thanks to the continuous technical developments, research has now moved to a new and more complex level: major efforts are being invested in identifying novel advanced cardioprotective strategies aiming to modify the genetic profile and the function of cells involved in the early infarct expansion.^2^ The efficacy of such complex and expensive treatments will also depend on the availability of accurate quantitative diagnostic techniques, such as cardiovascular magnetic resonance (CMR).^3^ CMR imaging is considered the gold standard noninvasive imaging technique for myocardial tissue characterization and quantification of acute and chronic myocardial injury after MI. However, late gadolinium enhancement (LGE) for the assessment of acute necrotic myocardium^4^ is partly hampered by methodological and technical challenges.^5,6^ Parametric mapping methods are becoming the techniques of choice for quantitative voxel-based tissue characterization postrevascularization because of their major diagnostic accuracy.^7–9^ During ischemia reperfusion, the deterioration of the microvascular function and endothelial structure are crucial determinants of the degree of cellular damage and water molecule exchange.^10^ Native T1 mapping techniques provide a quantitative biomarker of intracellular and extracellular environments of the myocardium without the need for intravenous contrast agents; native T1 mapping accurately depicts myocardial edema in the area at risk after an acute ischemic event.^11,12^ Furthermore, initial evidence would suggest that native T1 mapping might allow for assessment of severity of injury and prediction of recovery.^8^ The use of native T1 mapping to distinguish reversible (namely oedematous myocardium) and irreversible injury (namely necrotic myocardium as assessed by LGE) postacute MI without use of contrast, would be of major clinical use, in that it would not only be safer in patients with kidney impairment and would shorten the scan time (by avoiding contrast-based techniques) but would also potentially allow for an early accurate stratification of those acute patients in need of more aggressive treatment.^13^

**See Editorial by Garg and Plein**

**See Clinical Perspective**

We sought to investigate (1) whether acute native T1 mapping distinguishes reversible oedematous versus irreversible necrotic myocardial injury and (2) the predictive value of native T1 mapping for 6-month functional recovery over standard CMR measures of infarct size and microvascular function.

## Methods

### Patient Population

The study protocol was approved by the local ethics committee, and all patients gave written informed consent. Sixty ST-segment–elevation MI patients undergoing primary percutaneous coronary intervention (PPCI) within 12 hours of symptoms onset were prospectively enrolled as part of the OxAMI study (Oxford acute myocardial infarction). ST-segment–elevation MI was defined as per current guidelines.^14^ Acute clinical management reflected contemporary practice and guidelines (further details in the Data Supplement). Troponin I was assessed pre-PPCI (n=50) and at 6 (n=57), 24 (n=57) and 48 hours (n=52) post-PPCI; area under the curve was calculated using the trapezoidal rule to express this as summary measure of infarct size.^15^

### Cardiac Magnetic Resonance Protocol

CMR was performed on a 3 Tesla MR scanner (either MAGNETOM TIMTrio or MAGNETOM Verio; Siemens Healthcare, Erlangen, Germany) acutely (12–96 hours post-PPCI) and at 6 months. The CMR protocol (details in the Data Supplement) included cine, T2-prepared steady state free precession imaging, native shortened modified look-locker inversion recovery (ShMOLLI) T1 mapping, LGE, and first pass perfusion.

### CMR Imaging Analysis

Anonymized images were analyzed using cvi42 software (Circle Cardiovascular Imaging, Inc, Calgary, Canada) by experienced operators. LV function, segmental wall thickening (WT), quantification of edema, myocardial blood flow (MBF), and LGE were performed as described previously.^16^ The signal intensity threshold indicating myocardial edema was set at 2 SD above the remote reference region of interest (ROI); full width at half maximum technique was used on LGE.^17^ Microvascular obstruction (MVO) or hemorrhage were defined as the low-intensity core within an area of LGE or T2W imaging and were included in the reported areas of infarction or edema, respectively. Furthermore, LGE MVO was assessed separately and expressed as a percentage of total LV mass. LGE transmurality was assessed as described previously.^16^ T1 maps underwent strict and extensive quality control as described previously.^8^ Segmental T1 values were derived from short-axis T1 maps using in-house dedicated software MC-ROI (Interactive Data Language, version 6.1; Exelis Visual Information Solutions, Boulder, CO; Further details in the Data Supplement).

### Native T1 Analysis—ROI-Based Analysis: Derivation Cohort and Validation Cohort

Patients were randomly partitioned (with a ratio of 5:1) into 2 groups: a derivation cohort (n=50) for threshold identification and a validation cohort (n=10) for method validation and proof of concept as described previously.^18^

#### Derivation Cohort

**Figure 1.**
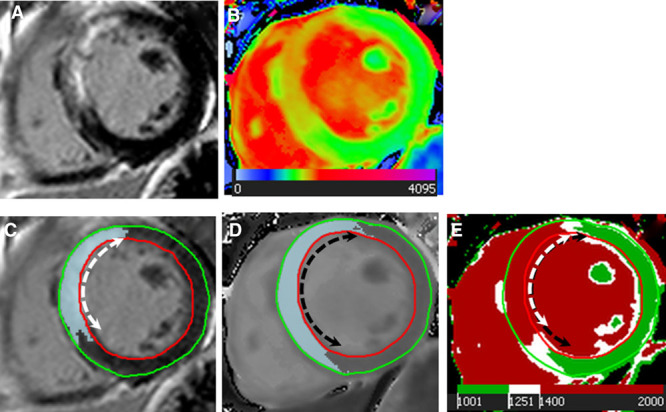
Region of interest (ROI)-based analysis for assessment of T1 thresholds. **A**, Acute late gadolinium enhancement (LGE) showing septal positive enhancement; (**B**) acute native T1 map showing increased septal T1s; (**C**) Full width half maximum quantification of corresponding LGE myocardium ROI; (**D**) quantification of oedematous myocardium ROI on grayscale native T1 shortened modified look-locker inversion recovery; (**E**) corresponding areas of edema and necrotic myocardium identified using T1 threshold values of 1400 ms (T1_irrev_) and 1250 ms (T1_rev_).

ROI-based T1 analyses were performed using cvi42 software on LV short-axis slices with no MVO or hemorrhage. To define the distribution of T1 values in areas of infarcted versus salvaged myocardium (defined as the oedematous regions adjacent to the LGE), ROIs were manually drawn on the threshold-enhanced area on acute LGE (ROI T1_LGEpos_) (Figure 1C) and transferred onto the corresponding acute matching ShMOLLI T1 map; edema was identified on T1 maps by using a threshold of 2 SD threshold above the remote normal myocardium.^8^ ROIs were then manually drawn on the salvaged myocardium (ROI T1_edema_). Anatomic landmarks ensured matching of the corresponding areas as published previously.^13^ The identified T1 values were used to establish cutoff thresholds for reversible (T1_rev_) and irreversible injuries (T1_irrev_).

Using the same principle, 6-month T1 values in the myocardium corresponding to the enhanced area of LGE 6-month could be identified (ROI T1_scar_).

#### Proof of Concept: Validation Cohort

Using the identified T1 thresholds, an expert MRI-trained cardiologist blinded to the LGE and edema analysis performed threshold-based analysis on ShMOLLI T1 maps on a different set of 10 patients to identify regions of irreversible injury on acute T1 maps (Figure 1E). If visually identified, MVO areas were included in the lesion size.

### Statistical Analysis

All analyses were performed using Matlab 2014a and R 3.2.3. Continuous variables were expressed as mean±SD (except where specified otherwise). Categorical variables were expressed as n (%). Normality was checked by Shapiro–Wilks test. Student *t* test (paired and unpaired) was used for comparison of continuous variables. Nonparametric Kruskal–Wallis with Dunn test was used to (1) compare differences in mean T1 values in in predefined ROIs (ROI T1_LGEpos_ versus ROI T1_edema_ versus ROI T1_remote_) and (2) assess the independence of T1 acute on the degrees of LGE transmurality (<50%, 50% to 75%, >75%) because normality of data was violated. One-way ANOVA with Tukey–Kramer test was used to compare multiple means of segmental MBF values in 3 subgroups partitioned according to T1 cutoff values as reversible injury (T1_rev_), irreversible injury (T1_irrev_), and remote (T1_remote_), when data normality and homogeneity of variances were met. Max-*t* test for multiple comparisons adjusted for unequal variances was adopted to compare (1) segmental 6-month WT in subgroups split according to T1 cutoff values and (2) multiple T1 means in the ROIs of acute T1_LGEpos_, 6-month T1_LGEpos_, acute T1_remote_, as heteroscedasticity was observed. Correlation was expressed as Pearson correlation coefficient. Bonferroni method was adopted to adjust *P* values in the Dunn test to control family-wise error rate. Classification tree analysis was adopted to detect T1 thresholds. The criteria for validation of the T1 thresholds is given in the Data Supplement. Exhaustive best-subset predictor selection approach^19^ was used to determine the best multivariable linear regression model to predict 6-month WT based on 3 predictors, that is, MBF, LGE, and T1 mapping at 24 hours. The relative importance of predictors (summing to 100%) was assessed by relative importance metric of covariate, LMG,^20^ which was calculated using R package relaimpo. Moreover, the multivariable logistic regression model was used to predict 6-month WT, using a 40% cutoff. Similarly, MBF, LGE, and T1 mapping at 24 hours were chosen as potential baseline independent variables. Receiver-operating characteristic analysis was performed to assess model performance of logistic regression.

## Results

Patient characteristics are given in Table 1. Seventy patients were screened: 10 were excluded (including 5 points where imaging protocol could not be completed because of patients’ lack of compliance and 5 points with breathing artifacts on acute T1 maps). One patient missed his follow-up scan at 6-month.

**Table 1.**
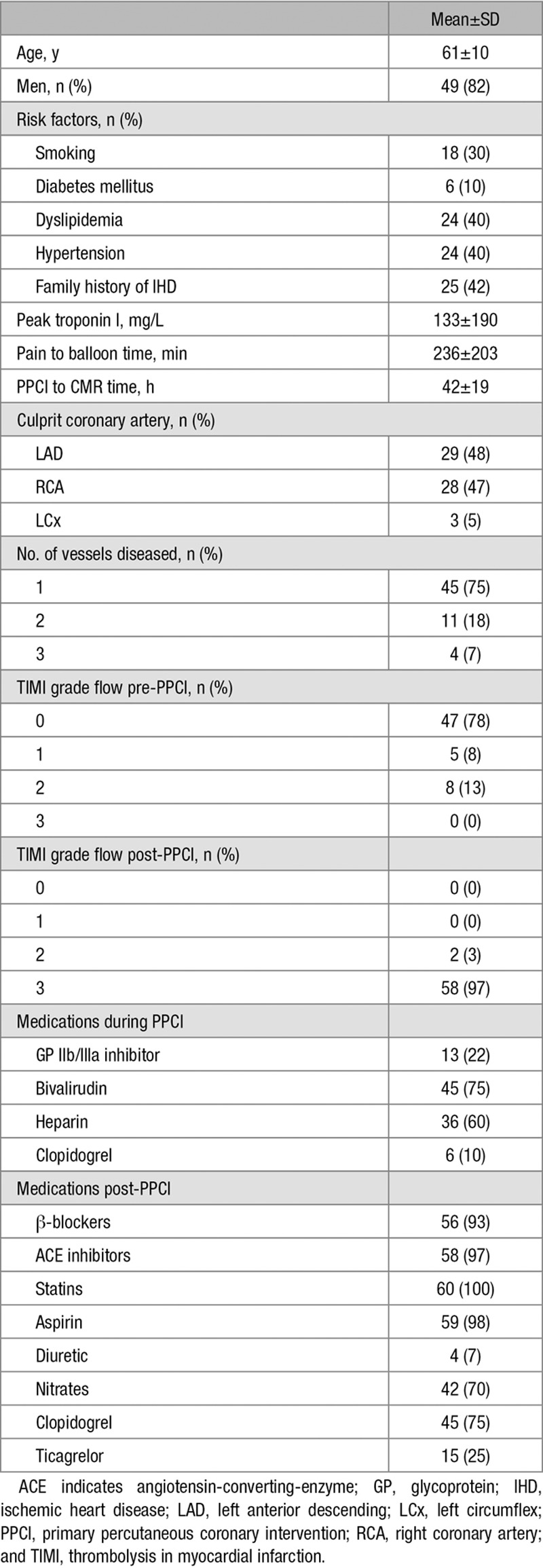
Baseline Characteristics of the Study Population

CMR findings are summarized in Table 2. There was no significant difference between measurements of the area of edema using T1 mapping and T2W (difference of 1.5±6.0% per slice, *P*=0.09, and 2.0±6.7% per-patient basis, *P*=0.38).

**Table 2.**
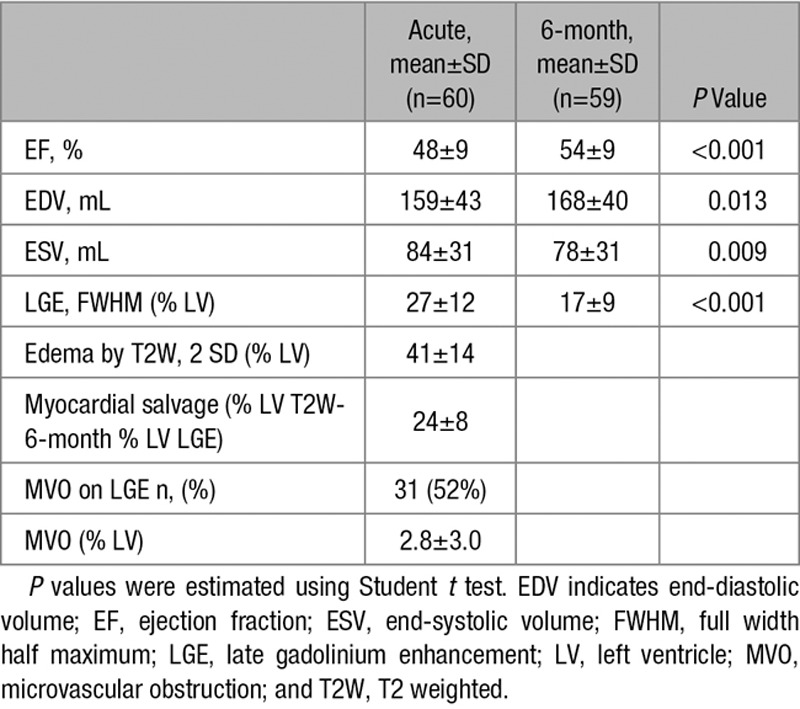
CMR Findings

### Acute T1 Native Mapping Predicts Reversible Versus Irreversible Myocardial Injury—ROI Analysis

For the ROI-based T1 analysis of reversible versus irreversible injury, 58 acute short-axis slices without MVO were available of which 43 had matching 6-month T1 maps.

**Figure 2.**
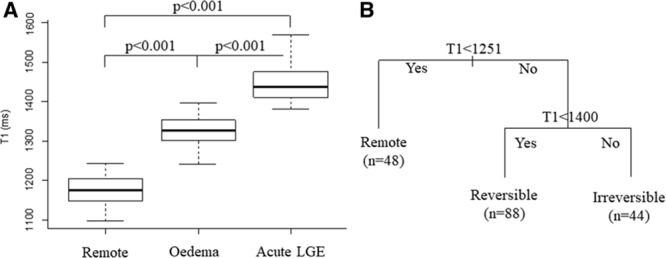
Distribution of T1 values and identification of T1 thresholds. **A**, Mean T1 values in the remote, oedematous, and late gadolinium enhancement (LGE) myocardium on the derivation cohort; (**B**) classification tree model was applied to identify T1 acute threshold values T1_rev_ and T1_irrev_.

ROI-based analysis on the derivation cohort was used to assess the acute native T1 values in areas of necrosis versus edema versus remote. T1 values were significantly different with T1_LGEpos_=1447±44 ms, T1_edema_=1327±36 ms, and T1_remote_=1177±34 ms (*P*<0.001; Figure 2A). By applying a decision tree model to the above derived T1s, the cutoff T1 values for oedematous (T1_rev_) versus remote (T1_remote_) and oedematous versus necrotic myocardium (T1_irrev_) were identified as 1251 and 1400 ms, respectively (Figure 2B), with a prediction accuracy of 96.7 (95% confidence interval, 82.8% to 99.9%) with sensitivity 100%, 100%, and 91% and specificity 100%, 95%, and 100% for normal tissue, myocardial edema, and necrotic myocardium, respectively.

**Figure 3.**
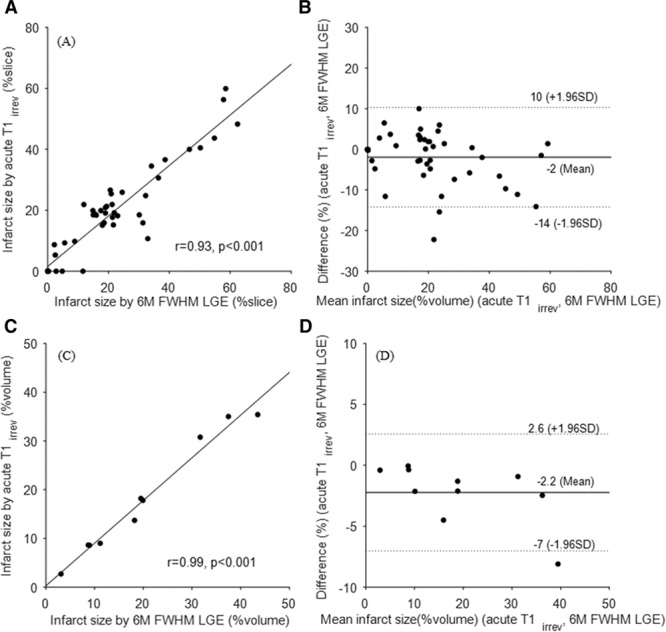
Linear regression and Bland–Altman plots for comparison between acute irreversible volume by T1_irrev_ threshold and 6-month FWHM late gadolinium enhancement (LGE). **A** and **B**, Per slice based analysis show a mean difference of ~2% and intraclass correlation ICC=0.92 with 95% confidence interval (CI; 0.86–0.95). **C** and **D**, Per patient-based analyses on matched volume show a mean difference of ~2% and ICC=0.97 with 95% CI (0.86–0.99). ICC indicates intraclass correlation coefficient; and FWHM, full width at half maximum.

By using the proposed T1_irrev_ threshold on the validation cohort, the volume of irreversibly damaged tissue was in good agreement with the 6-month LGE volume, both on a slice by slices basis (*r*=0.93; n=47; Figure 3A and 3B), and on per-patient basis (*r*=0.99; n=10; Figure 3C and 3D).

### Irreversible Injury Assessed by T1_irrev_ Acute Cutoff Correlates With Area Under the Curve TnI Post-PPCI and 6-Month Ejection Fraction

**Figure 4.**
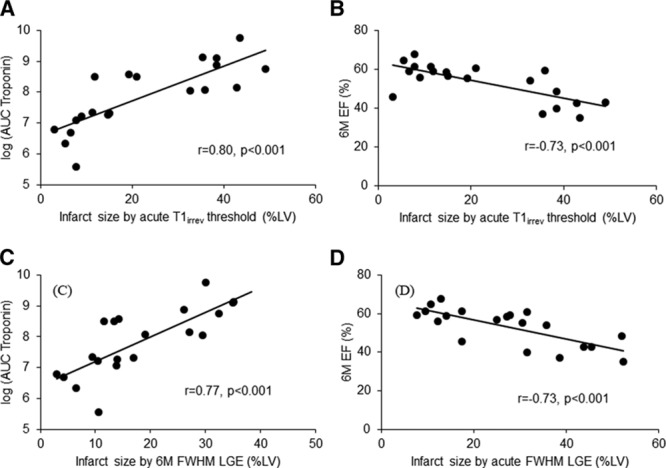
Clinical validation of native T1_irrev_ threshold. Correlation between the volume of irreversibly damaged myocardium (as assessed by native acute T1_irrev_ threshold or acute FWHM late gadolinium enhancement [LGE] or 6-month FWHM LGE) and the log area under the curve (AUC) of the troponin I (**A** and **C**) and the 6-month ejection fraction (EF; **B** and **D**). FWHM indicates full width at half maximum technique; and LV, left ventricular.

Troponin I measurements at 4 time points were available in 46 patients, of which 20 patients had full LV T1 map coverage. For these 20 patients, there was a strong correlation between the volume of myocardial damage using acute T1_irrev_ as threshold and log area under the curve troponin (*r*=0.80; Figure 4A and 4C). The correlation between 6-month ejection fraction and the amount of LV damage as assessed by acute T1_irrev_ cutoff and LGE acute was strong (*r*=−0.73; Figure 4B and 4D).

### The Healing of Myocardial Injury as Assessed by Changes in T1 Values From Acute to 6 Months, and Its Relationship to LGE and Resting MBF—Segment-Based Analysis

**Figure 5.**
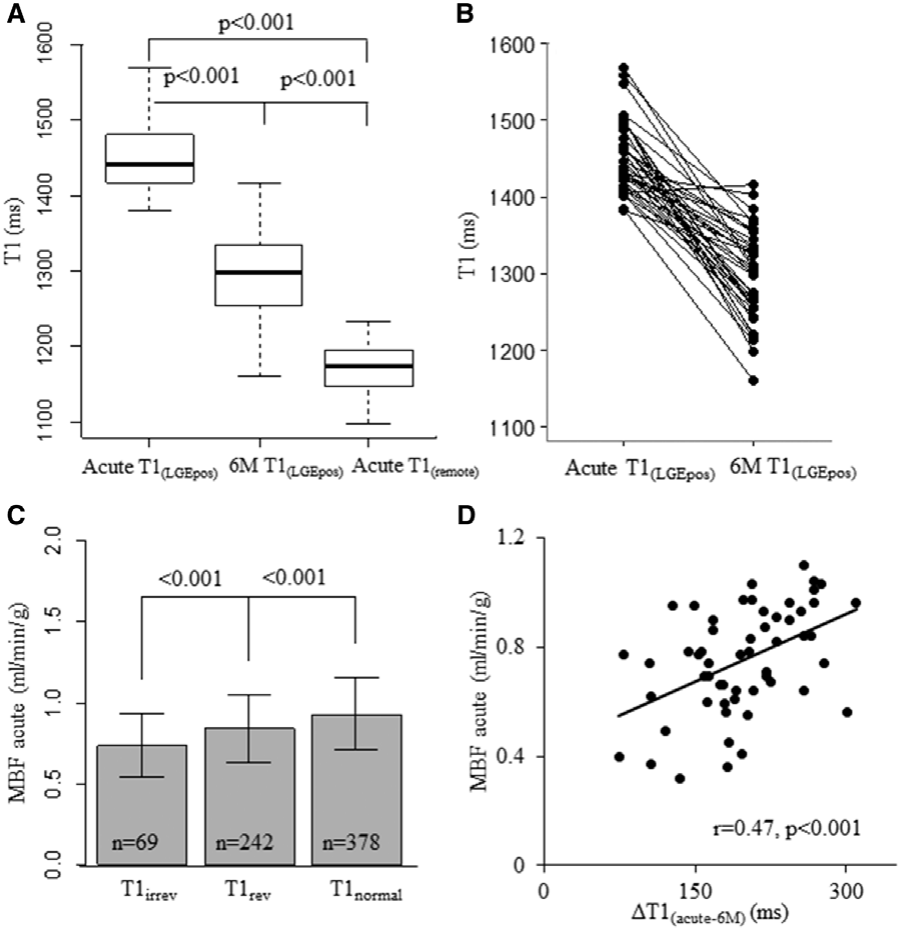
T1 change from acute to 6 months, and its relation with myocardial blood flow (MBF) in nonmicrovascular obstruction segments. **A**, on 43 matched slices 6-month T1_LGEpos_ is significantly lower than acute T1_LGEpos_ but still higher than acute T1_remote_; (**B**) the change in T1 values is given for each measurement; (**C**) acute MBF is significantly different in T1_irrev_ vs T1_rev_ vs T1_normal_ segments; (**D**) the segmental change of T1 from acute to 6 months ΔT1_LGEpos acute-6 months_ significantly relates to the MBF.

To determine the changes in T1 from the acute to chronic stage, we assessed T1 values in the ROIs corresponding to the scarred myocardium as defined by full width at half maximum threshold analysis both in acute and at 6 months. The acute ROI T1_LGEpos_ values were significantly higher than the 6-month ROI T1s (acute T1_LGEpos_=1451.7±45.9 ms; 6-month T1_scar_=1294.4±58.7 ms; *P*<0.001; Figure 5A and 5B), independent of the degree of LGE transmurality (Figure I in the Data Supplement); the relative reduction in ROI T1_LGEpos_ values (ΔT1_LGEpos acute-6 months_) was 11±5% (range, −1% to 20%); 6 mol/L T1s were also significantly higher than the T1 values depicted in the remote myocardium (T1 remote myocardium=1173.4±34.2 ms; *P*<0.001; Figure 5A).

To assess whether the acute microvascular function played a role in the myocardial healing post-MI as defined by the T1 changes over time, we first assessed the differences in acute MBF in myocardial segments reversible versus irreversible injury (defined by T1_rev_ and T1_irrev_ thresholds). In non-MVO segments, MBF was significantly different between T1_irrev_, T1_rev_, and T1_remote_ segments (MBF=0.74±0.20 ml/min/g versus 0.85±0.21 ml/min/g versus 0.93±0.22 ml/min/g, respectively; *P*<0.001; Figure 5C). Overall, there was a significant negative weak correlation between segmental MBF and segmental T1 (*r*=−0.30) in acute MI; furthermore, in T1_irrev_ segments, the magnitude of change in T1 values from acute to 6 months was greater in segments with less-impaired acute MBF (*r*=0.47; Figure 5D). In segments with MVO, MBF was significantly lower than in non-MVO T1_irrev_ segments (MBF=0.59±0.18 mL/min/g versus 0.74±0.20 mL/min/g; *P*<0.001).

### Acute T1 Values Predict Functional Recovery at 6 Months Over Standard CMR Techniques

**Figure 6.**
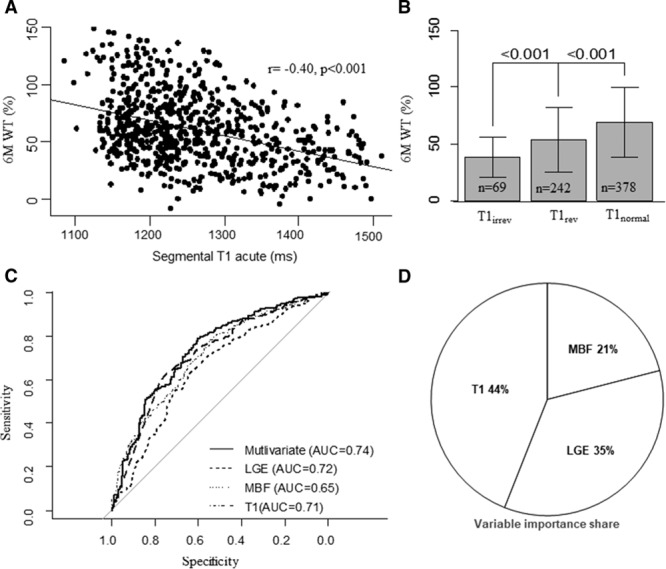
Acute T1 and 6-month wall thickening (WT) in nonmicrovascular obstruction segments: relationship and predictive value. **A**, Moderate negative correlation between acute native T1s and 6-month WT (*r*=−0.40; n=689). **B**, 6-month WT (%) values are given in myocardium defined by T1_normal_, T1_rev_, and T1_irrev_ (69.0±30.3% vs 53.8±28.1% vs 38.5±17.6%; *P*<0.001). **C**, Receiver-operating characteristic curves for multivariable logistic regression using a 40% cutoff for dependent variable WT 6 months. Area under the curve for T1 mapping and late gadolinium enhancement (LGE) are similar and significantly better than myocardial blood flow (MBF). **D**, Relevant variable importance share to define the contribution of each predictor to the variation in 6-month WT (total explained WT variance ~20%) assessed using the LMG importance score (Data Supplement) in the multivariable linear regression: the T1 mapping contributes more to the prediction of WT compared with LGE and MBF.

In non-MVO segments, a moderate negative relationship between the 6-month WT and acute T1 values was found, using both segmental (*r*=−0.40) and threshold-based analysis (*P*<0.001; Figure 6A and 6B).

Receiver-operating characteristic curves using a 40% cutoff for WT 6 months showed that T1 mapping and LGE were equally good predictors of 6-month WT (Figure 6C). By applying multivariable linear regression and variable importance share analysis (Figure 6D), results showed that the proportion to which T1 mapping accounts for the total explained WT variance is greater than LGE or MBF (44% versus 35% versus 21%, respectively). Furthermore, multivariable linear regression suggests that an increase in T1 values of 100 ms is significantly associated with a decrease in WT of 8% (95% confidence interval, 5% to 11%; *P*<0.001).

## Discussion

We explored the ability of acute native T1 mapping to distinguish reversible versus irreversible injury without the use of contrast media. Furthermore, we assessed the predictive value of T1 mapping as determinants of long-term LV functional recovery compared with standard CMR techniques. Our study has several main findings: (1) acute native ShMOLLI T1 mapping allows accurate assessment of irreversibly injured myocardium, (2) T1 values decrease from acute to 6-month but remain higher than the remote allowing for assessment of scar at 6-month, (3) the severity of acute injury as expressed by increasingly higher T1 values, and the degree of myocardial healing assessed by change in T1 values from acute to 6 months, are associated to the acute microvascular function (MBF), (4) the amount of acute myocardial irreversible injury assessed using native T1 mapping correlates strongly with TnI and with 6-month ejection fraction, (5) native T1 mapping is a strong predictor of 6-month LV remodeling when compared with standard CMR techniques, such as MBF and LGE.

### Native T1 Mapping at Acute Stage Post-MI Predicts Irreversibility of Injury

LGE is the clinical standard to assess scar volume in stable coronary artery disease.^21^ In the acute setting, LGE depicts myocardial necrosis,^4^ but its accuracy and hence clinical applicability has been repeatedly challenged.^5,6,22^ Careful timing of image acquisition postcontrast administration and careful postprocessing using stringent thresholds (such as full width at half maximum or 5 SD) are critical to avoid overestimation of the infarct size because of increased extracellular space in the peri-infarct zone.^23,24^ By determining voxel-wise T1 values on a continuous scale as measurements of the tissue composition, native T1 mapping could potentially not only overcome the LGE limitations described above but also provide additional information on the severity of injury rather than just a volume of injury.^8^ Its additional diagnostic value in quantifying ischemic myocardial oedema^12^ and the area at risk^7^ has been established. Pathology studies have demonstrated increasingly high T1 values in relation to the duration of ischemia^25^ and the consequent intra and extracellular molecular changes.^26,27^ We previously demonstrated in a small cohort that elevated segmental T1 values significantly correlate to the transmural extent of 6-month LGE.^8^ However, the use of segmental analysis averages out the T1 values and hence lessens the effectiveness and clinical applicability of a voxel-wise quantitative technique. For the first time, the current new study provides a T1 cutoff value to differentiate edema (reversible injury) from more severe irreversible myocardial damage as depicted by full width at half maximum LGE at 24 hours postacute MI. Our data also show a strong correlation between the volume of irreversibly damaged myocardium as assessed by T1 and (1) the log area under the curve of the troponin I and (2) the ejection fraction at 6 months.

### Changes in T1 Values From Acute to 6 Months

To assess the additional predictive value of T1 mapping compared with standard acute CMR imaging measures of infarct size and microvascular function, we first assessed the relationship between the changes in T1 values over time with LGE transmurality and MBF. Not only our results are consistent with previously published data demonstrating higher 6-month T1 values in scarred myocardium compared with remote,^16^ in addition, we demonstrate a change in T1 values from acute to 6 months irrespective of the LGE transmurality. These findings validate the clinical use of native T1 values to detect irreversible myocardial injury acutely and most importantly distinguish it from chronically scarred myocardium. Furthermore, in patients with no evidence of MVO at LGE, we show a strong association between the microvascular dysfunction post-PPCI and the higher T1 values and the magnitude of T1 change from acute to chronic.

MBF is a critical determinant of myocardial healing and an important target at the time of reperfusion to improve outcomes, as we have shown previously.^28^ Previous pathology studies showed the existence of a delayed microvascular impairment,^29^ happening hours after reperfusion because of the accumulation of erythrocytes and neutrophils^29^ and different to the immediate no-reflow characterized by severe capillary damage with coagulation necrosis.^30^ Carrick et al^9^ recently reported a progressively higher detection of myocardial hemorrhage using T2* mapping from the early hours to first days after MI. It is plausible that even in patients with no evidence of immediate no-reflow as shown by LGE, the microvascular function deteriorates in the following 24 hours, leading to an inefficient myocardial healing process affecting LV remodeling.

### The Additional Predictive Value of 6-month Functional Recovery of Native T1 Mapping Compared With LGE and MBF

We show that irreversibly injured myocardium as detected by T1 values shows the least WT at 6 months; both LGE and T1 mapping are strong predictors of 6-month recovery in agreement with previously published data; however, our results show that T1 has a more relative importance as predictor of WT compared with LGE. These findings have important clinical significance. Currently, there is considerable uncertainty on which standard imaging marker (ie, infarct size, MVO, and salvaged myocardium) carries the most prognostic weight. Establishing a valid predictor of long-term remodeling using the mere assessment of volumes of injury as biomarkers might be challenging because of the rapid changes happening in the tissue composition in the early hours post-PCI.^31^ Mapping techniques offer the potential additional advantage of quantifying exactly the expected change in long-term function based on the increase in acute T1 values. As such, T1 mapping would allow for an accurate stratification of patients who might need more aggressive or additional treatment to prevent remodeling. In a time where efforts are dedicated to developing novel therapies targeting the immune system early after the onset of ischemia, such additional diagnostic accuracy would play a key role in tailoring personalized treatments.

### Study Limitations

The established T1 estimates and thresholds refer specifically to ShMOLLI T1 mapping technique at 3T scanners. Appreciable differences can be expected for other T1 mapping sequences and at different magnetic field strength. This is a proof of concept study that demonstrates the feasibility of T1 mapping to differentiate tissue and predict remodeling assessed by surrogate end points, in mostly single vessel disease ST-segment–elevation MI patient. In this cohort, we have not assessed extracellular volume because this requires postcontrast T1 mapping and was perceived too challenging for imaging the MI patients in acute setting. Extracellular volume plays a crucial role in acute MI and healing of the myocardium. Confirmatory studies on larger populations will be needed to assess the validity and applicability of our findings to all mapping methods at different fields strengths/vendors and to exploit the full clinical predictive value.

## Conclusions

Native ShMOLLI T1 mapping performed in the early hours post-MI allows accurate assessment of the severity of myocardial damage, and it differentiates reversible and irreversible injury. T1 mapping values are a stronger predictor of LV remodeling at 6 months compared with LGE. ShMOLLI T1 mapping could represent a more accurate and faster noncontrast alternative to standard CMR LGE imaging in acute ST-segment–elevation MI patients.

## Acknowledgments

We thank the clinical staff in the Oxford Heart Centre and Oxford Centre for Clinical Magnetic Resonance Research; Dr Paula Colmenero, Juliet Semple, Peter Manley, Carol Davey, and Lisa Gaughran in the Oxford Acute Vascular Imaging Centre for their expertise and work in the coordination of the OxAMI study (Oxford acute myocardial infarction) supported by the Oxford National Institute for Health Research–Oxford Biomedical Research Centre; and Prof Paul Baxter in Leeds Institute of Health Sciences for his advice on statistical analysis.

## Sources of Funding

This work is supported by the British Heart Foundation and the Oxford National Institute for Health Research Biomedical Research Centre. Profs Choudhury, Channon, and Neubauer acknowledge support from the Oxford British Heart Foundation Centre of Research Excellence. Dr Dall’Armellina is a BHF Intermediate Clinical Research Fellow. Dr Dawkins is a BHF Oxford Centre of Research Excellence Clinical Research training fellow. Dr Liu is supported by the BHF.

## Disclosures

None.

## Supplementary Material

## CLINICAL PERSPECTIVE

Prognosis after acute myocardial infarction is primarily dictated by the degree of irreversible myocardial injury and by left ventricular remodeling. Future developments in identifying the efficacy of cardioprotective strategies will also depend on the availability of accurate diagnostic techniques. The applicability of standard late gadolinium enhancement techniques in the acute setting might be challenging because of the increased extracellular space and the well-known dynamic changes happening in the myocardium in the early hours of the healing stage. CMR mapping is becoming the technique of choice for quantitative voxel-based tissue characterization postrevascularization because of its major diagnostic accuracy. Such techniques offer a major potential advantage in allowing the assessment of the severity of injury rather than only a binary quantification of the injured volume and by doing so overcoming the challenges of late gadolinium enhancement in acute myocardial infarction. This study defines a cutoff value for native T1 mapping to distinguish reversible versus irreversible injury in acute myocardial infarction patients without need for contrast; furthermore, it shows the potential predictive value for long-term remodeling of acute T1 values. The translational clinical value of such findings is major in that it allows using a single scan acquisition to derive a biomarker relevant for risk assessment without need for multiple prolonged acquisitions or administration of contrast.
