# Supplementary material for: CMR Native T1 Mapping Allows Differentiation of Reversible Versus Irreversible Myocardial Damage in ST-segment–Elevation Myocardial Infarction: An OxAMI Study (Oxford Acute Myocardial Infarction)
Source: Circ Cardiovasc Imaging. 2017 Aug 10;10(8):e005986. doi: 10.1161/CIRCIMAGING.116.005986 (PMC5555391; doi:10.1161/CIRCIMAGING.116.005986)
Supplement: Supplementary file 1 [file hci-10-e005986-s001.docx]

**Supplemental Material**

**Patient population**

Diagnosis of STEMI required chest pain lasting longer than 30 min, within 12 h from onset of symptoms, and ST-segment elevation of ≥2 mm (0.2 mV) in at least 2 contiguous leads on ECG. Acute clinical management was at the discretion of the responsible physician, with the intention to reflected contemporary practice and guidelines.[^1^](#_ENREF_1) Exclusion criteria were presentation with cardiogenic shock, hemodynamic instability lasting >12 hours after revascularization, previous myocardial infarction, previous revascularization procedure (coronary artery bypass grafts or PCI), severe heart valve disease, cardiomyopathy, contraindications to CMR.

**CMR acquisition**

Matching short axis slices covering the LV were acquired using an established CMR protocol[^2^](#_ENREF_2)^,^[^3^](#_ENREF_3) including: cine, T2-prepared SSFP imaging, native Shortened Modified Look-Locker Inversion recovery (ShMOLLI) T1 mapping, and late gadolinium enhancement (LGE). In addition 3 to 5 short-axis images were acquired using First Pass Perfusion (FPP) imaging.Using T1 weighted FPP imaging, 3 to 5 short-axis images were acquired every heart beat to track the first pass of a gadolinium-based contrast agent (0.03 mmol/kg; gadoterate meglumine, Dotarem, Guerbet, Villepinte, France) injected at rest. LGE images were collected 10-15 min after the administration of 0.1 mmol/kg contrast agent (gadoterate meglumine, Dotarem, Guerbet, Villepinte, France). The inversion time was adjusted for optimal nulling of remote normal myocardium.

T2W was performed using a T2-prep-SSFP single shot sequence with surface coil correction (TE/TR = 1/4.1 msec; effective TE = 60 msec; flip angle = 90°; voxel size: 2.1 × 1.6 × 8 mm). ShMOLLI T1 maps were generated from 5-7 SSFP images with variable inversion preparation time as described previously.[^4^](#_ENREF_4) Typical acquisition parameters were: TE/TR = 1.07/2.14 msec, flip angle=35°, FOV=340×255mm, matrix size=192×144, 107 phase encoding steps, actual experimental voxel size = 1.8 × 1.8 × 8 mm, interpolated reconstructed voxel size = 0.9 × 0.9 × 8 mm, GRAPPA = 2, 24 reference lines, cardiac delay time TD = 500 msec and 206 msec acquisition time for single image, phase partial Fourier 6/8.[^4^](#_ENREF_4) LGE was performed with a T1-weighted segmented inversion recovery gradient echo-phase sensitive-inversion recovery (GRE_PSIR) sequence (TE/TR = 2.5 msec/5 msec, voxel size 1.8 × 1.4 × 8 mm, flip angle 20°). Images were collected 10-15 min after the administration of 0.1 mmol/kg contrast agent (gadoterate meglumine, Dotarem, Guerbet, Villepinte, France). The inversion time was adjusted for optimal nulling of remote normal myocardium. SSFP cine images were acquired using retrospective gating (TE/TR = 1.4/3.2 msec; flip angle = 50°; voxel size: 1.6 × 1.6 × 8 mm). Two to three-fold accelerated parallel imaging (GRAPPA) was used to shorten the breath-hold.

For first pass perfusion (FPP) imaging, an ECG-gated T1-weighted (saturation-recovery prepared) fast gradient echo sequence was used (echo time, 1.04 msec; repetition time, 2 msec, saturation recovery time, 100 msec; voxel size, 2.1×2.6×8 mm3; flip angle, 17°) to acquire 3 to 5 short-axis images every heart beat to track the first pass of a gadolinium-based contrast agent (0.03 mmol/kg; gadoterate meglumine, Dotarem, Guerbet, Villepinte, France) injected at rest.

**CMR image analysis**

Infarct size and myocardial oedema were expressed as a percentage of total LV mass; myocardial salvage was derived by subtracting the percentage infarct size from the percentage of myocardial oedema.[^2^](#_ENREF_2)

Segmental quantitative perfusion analysis was performed using an in-house Matlab software as previously described.[^5^](#_ENREF_5) Absolute myocardial blood flow (MBF) in ml/min/g was calculated for each myocardial segment by Fermi-model constrained deconvolution of myocardial signal intensity curves with the arterial input.[^6^](#_ENREF_6)^,^[^7^](#_ENREF_7) MBF was corrected for the heart rate –systolic blood pressure product.[^8^](#_ENREF_8)^,^[^9^](#_ENREF_9)

*Native T1 analysis- segment-based analysis*

T1 maps underwent strict and extensive quality control as previously described[^3^](#_ENREF_3) by investigating the native T1 maps, raw T1 images, R^2^ maps, considering off-resonance artefacts, partial volume effect, poor T1 fit on the R^2^ maps, patient movement or low SNR.[^10^](#_ENREF_10) Apical slices affected by partial volume effects and slices where the LV outflow tract was visible, were excluded from the study.

Segmental T1 values were derived from short-axis T1 maps which were manually contoured to outline the endocardial and epicardial borders using in-house dedicated software MC-ROI (Interactive Data Language, version 6.1, Exelis Visual Information Solutions, Boulder, Colorado).

**Statistical analysis**

All analyses were performed using Matlab 2014a and R 3.2.3. Data normality was checked using Shapiro-Wilks test. The homogeneity of variance of data was evaluated using the Levene’s test.

Student’s t-test was used for comparison of continuous variables (EF, EDV, ESV and LGE) measured at 2 time points (paired) and for variable MBF between two groups at a single time point (unpaired). P-values less than 0.05 were considered statistically significant. Correlation was expressed as Pearson's product moment correlation coefficient.

Using 80% of the ROIs from the acute scans in the derivation cohort as a training set, a classification tree model optimized using 10 fold cross-validation for tree complexity, was adopted to determinate acute T1 cut-off values for remote tissue, reversible, and irreversible injury.[^11^](#_ENREF_11)^,^[^12^](#_ENREF_12) The prediction accuracy of such T1 values was assessed on the remaining 20% ROIs in the derivation cohort.[^13^](#_ENREF_13) Bland-Altman analysis was used to assess agreement of volume percentage of irreversible damaged tissue identified on LGE 6M versus threshold based acute T1 mapping analysis in validation cohort.

**Figure S1.** **T1 comparison with LGE transmurality**. Mean T1_(LGEpos)_ ROIs is independent of mean acute LGE transmurality (p=0.89).

**References**

1. O'Gara PT, Kushner FG, Ascheim DD, et al. 2013 ACCF/AHA guideline for the management of ST-elevation myocardial infarction: a report of the American College of Cardiology Foundation/American Heart Association Task Force on Practice Guidelines. Circulation 2013;127:e362-425.

2. Dall'Armellina E, Karia N, Lindsay AC, et al. Dynamic Changes of Edema and Late Gadolinium Enhancement after Acute Myocardial Infarction and Their Relationship to Functional Recovery and Salvage Index. Circulation: Cardiovascular Imaging 2011;4:228-36.

3. Dall'armellina E, Piechnik SK, Ferreira VM, et al. Cardiovascular magnetic resonance by non contrast T1 mapping allows assessment of severity of injury in acute myocardial infarction. Journal of cardiovascular magnetic resonance : official journal of the Society for Cardiovascular Magnetic Resonance 2012;14:15.

4. Piechnik S, Ferreira V, Dall'Armellina E, et al. Shortened Modified Look-Locker Inversion recovery (ShMOLLI) for clinical myocardial T1-mapping at 1.5 and 3 T within a 9 heartbeat breathhold. Journal of Cardiovascular Magnetic Resonance 2010;12:69.

5. Selvanayagam JB, Jerosch-Herold M, Porto I, et al. Resting Myocardial Blood Flow Is Impaired in Hibernating Myocardium: A Magnetic Resonance Study of Quantitative Perfusion Assessment. Circulation 2005;112:3289-96.

6. Jerosch-Herold M, Wilke N, Stillman AE. Magnetic resonance quantification of the myocardial perfusion reserve with a Fermi function model for constrained deconvolution. Med Phys 1998;25:73-84.

7. Jerosch-Herold M, Ravi Teja S, Cory MS, Norbert MW, Arthur ES. Analysis of myocardial perfusion MRI. Journal of Magnetic Resonance Imaging 2004;19:758-70.

8. Czernin J, Muller P, Chan S, et al. Influence of age and hemodynamics on myocardial blood flow and flow reserve. Circulation 1993;88:62-9.

9. Selvanayagam JB, Cheng ASH, Jerosch-Herold M, et al. Effect of Distal Embolization on Myocardial Perfusion Reserve After Percutaneous Coronary Intervention: A Quantitative Magnetic Resonance Perfusion Study. Circulation 2007;116:1458-64.

10. Ferreira VM, Piechnik SK, Dall'armellina E, et al. Non-contrast T1-mapping detects acute myocardial edema with high diagnostic accuracy: a comparison to T2-weighted cardiovascular magnetic resonance. Journal of cardiovascular magnetic resonance : official journal of the Society for Cardiovascular Magnetic Resonance 2012;14:42.

11. James G, Witten D, Hastie T, Tibshirani R. An Introduction to Statistical Learning: with Applications in R: Springer Publishing Company, Incorporated; 2014.

12. Willis SR, Ahmed HU, Moore CM, et al. Multiparametric MRI followed by targeted prostate biopsy for men with suspected prostate cancer: a clinical decision analysis. BMJ Open 2014;4:e004895.

13. Korteweg T, Tintore M, Uitdehaag BM, et al. A search for new MRI criteria for dissemination in space in subjects with a clinically isolated syndrome. Eur Radiol 2009;19:2244-8.
